# Molecular structure input on the web

**DOI:** 10.1186/1758-2946-2-1

**Published:** 2010-02-02

**Authors:** Peter Ertl

**Affiliations:** 1 Novartis Institutes for BioMedical Research, Novartis Campus, CH-4056 Basel, Switzerland

## Abstract

A molecule editor, that is program for input and editing of molecules, is an indispensable part of every cheminformatics or molecular processing system. This review focuses on a special type of molecule editors, namely those that are used for molecule structure input on the web. Scientific computing is now moving more and more in the direction of web services and cloud computing, with servers scattered all around the Internet. Thus a web browser has become the universal scientific user interface, and a tool to edit molecules directly within the web browser is essential.

The review covers a history of web-based structure input, starting with simple text entry boxes and early molecule editors based on clickable maps, before moving to the current situation dominated by Java applets. One typical example - the popular JME Molecule Editor - will be described in more detail. Modern Ajax server-side molecule editors are also presented. And finally, the possible future direction of web-based molecule editing, based on technologies like JavaScript and Flash, is discussed.

## Introduction

A program for the input and editing of molecules is an indispensable part of every cheminformatics or molecular processing system. Such a program is known as a molecule editor, molecular editor or structure sketcher. Its function is to facilitate entry of molecules or reactions into an computer with help of mouse and keyboard actions. There are two types of molecule editors: 3D editors, supporting creation of 3D molecular structures, mainly for use in molecular modeling applications, and 2D editors generating "flat" 2D molecule representations used as input to various molecular processing services, such as searches of chemical databases or the creation of chemical illustrations.

In this overview only 2D molecule editors used for chemical structure input on the web will be covered. The World Wide Web, introduced originally as a medium for exchange of scientific information, is affecting now practically all aspects of our life, but scientific and technical applications still benefit proportionally more from the web technology. Scientific computing is moving more and more in the direction of web services and cloud computing, with servers scattered all around the Internet, and the web browser becoming the universal scientific user interface. Chemistry is no exception from this trend and input of molecular structures directly within a web browser is therefore of utmost importance.

In this overview a history of entering molecules into web applications will be covered, starting from simple text entry boxes, continuing with the current situation dominated by Java applets and finally modern Ajax server-side molecule editors and future technologies, like pure client editors based on JavaScript, will be discussed. Only "true" platform independent web editors which can run in any standard web browser will be covered here. In addition to this type of tools, various molecular drawing programs are available also as so called plugins. These programs require relatively complex installation before use and are platform dependent, available in most cases only on Windows PCs. An overview of these types of molecule editors is available as a chapter in the cheminformatics textbook [1], as well as in an on-line review of chemical drawing programs [2].

## Early web-based structure input tools

**Figure 1:**
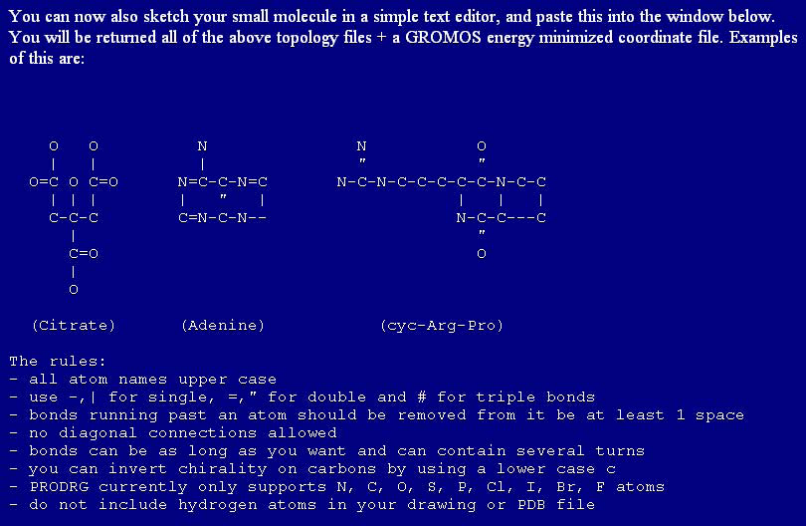
**Text-based structure entry notation used at the PRODRG2 server**.

The first web-based molecular processing tools did not have capability to enter molecule structures graphically, because the early web technology allowed only input of textual data. Users were therefore asked to input their molecules as SMILES strings [3] or to upload structure files created with help of locally installed molecule drawing programs. Several ingenious web developers tried to overcome this limitation by offering character-based or image-based construction of molecules from fragments, but these systems were not easy to use and therefore did not find general acceptance. Example of such an interesting system which allows construction of molecules from ASCII characters is still functional at the Dundee PRODRG2 modelling server [4] (Figure 1).

**Figure 2:**
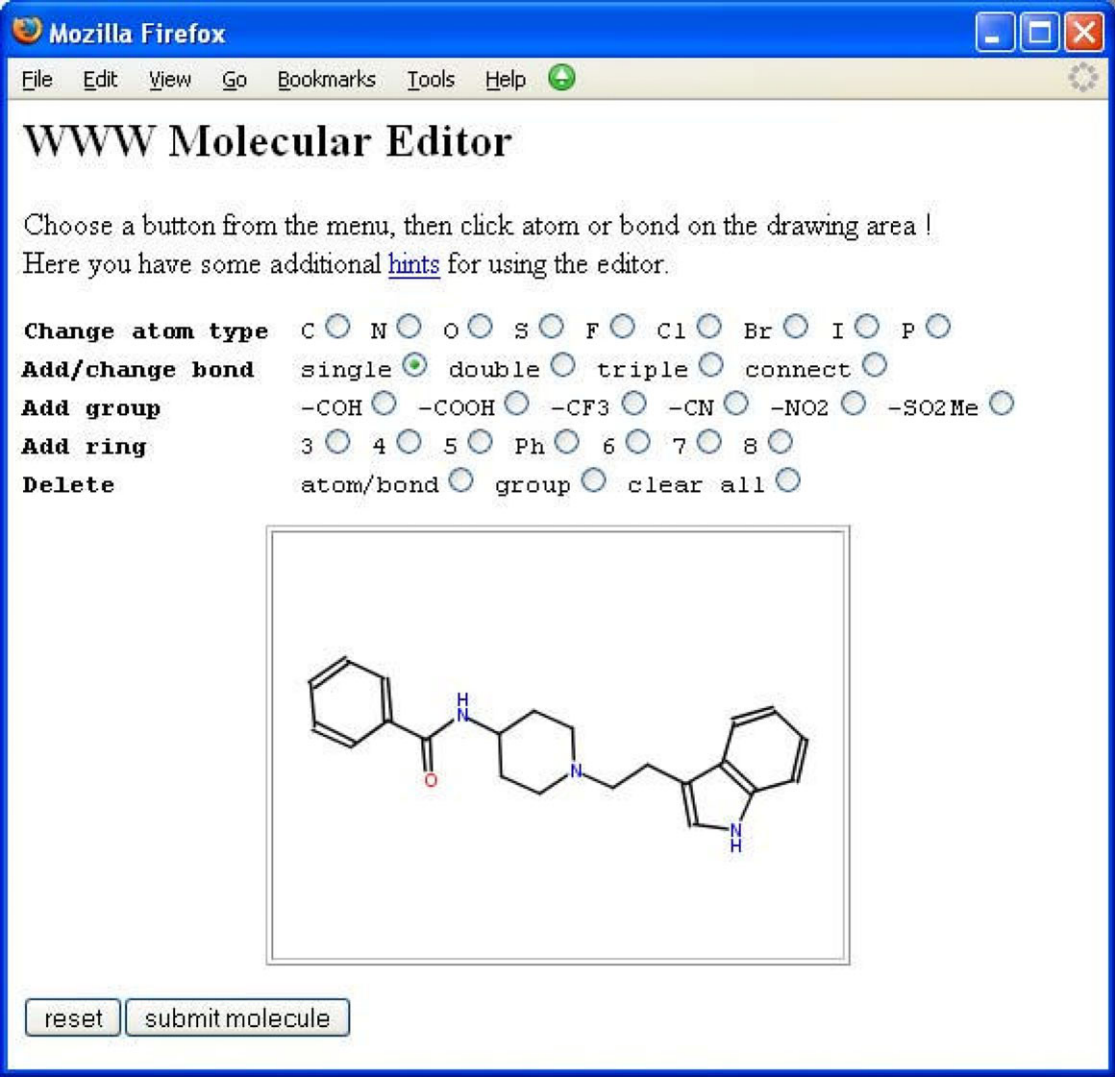
**Web-based molecule editor based on clickable map developed at Ciba-Geigy**.

**Figure 3:**
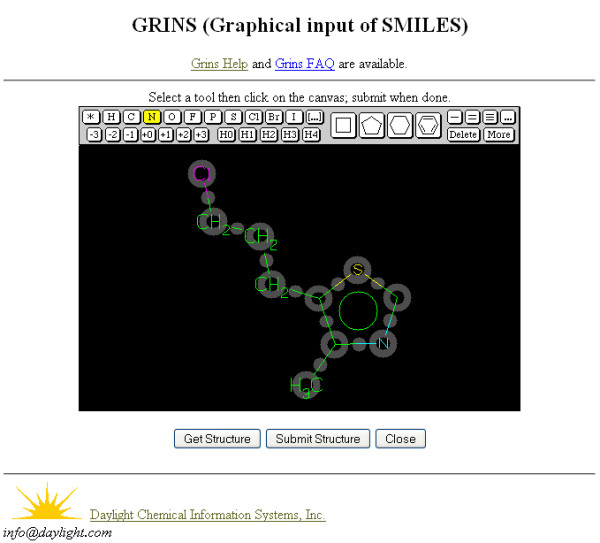
**Daylight GRINS editor**.

The first "true" web structure editor, which allowed creation and modification of molecules directly within a web page was developed at Ciba-Geigy (later Novartis) as part of the in-house web-based cheminformatics system [5] (Figure 2). This editor was based on so called "clickable map" technology. The structure to be modified was displayed on the web page as an image. The user had to select the required editing action from a menu and then click the atom or bond that should be modified. The click coordinates, together with the desired action, were sent to the server, where the structure was modified and a new molecular image was sent back. The disadvantage of this approach was that for every structural change, the program required a new connection to the server, where a native application had to be launched before responding to the client. Despite this, the editor was a huge step forward from previous text-only entry, enabling relatively easy interactive input of molecules on the web by medicinal chemists and it boosted the use of interactive cheminformatics services on the Ciba-Geigy intranet. This editor become direct inspiration to the Daylight GRINS editor [6] (Figure 3) based on the same principle.

## Java applets

**Figure 4:**
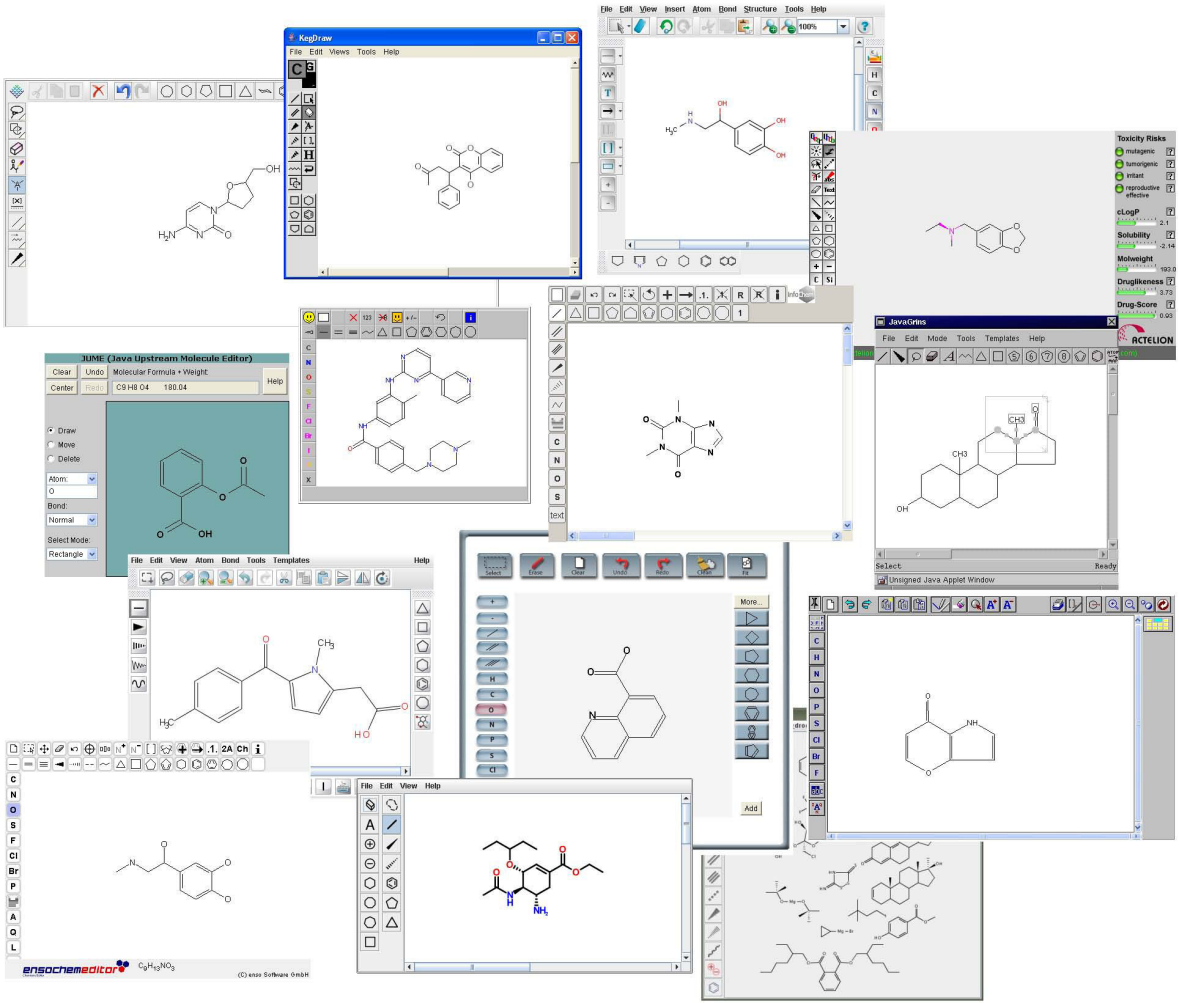
**Examples of molecule editor applets available on the Internet**.

Introduction of the Java programming language in 1995 contributed considerably to increased interactivity of web applications. Small graphical Java programs - applets - could be integrated directly into web pages to add practically any desired functionality. Probably the first molecule editor in Java was an applet written by D. Bayada from the Leeds University. Source code of this program was available and one can still find it on the Internet [7]. Independently a JME molecule editor applet was implemented at Ciba-Geigy (later Novartis). Many other molecule editor applets have been subsequently developed and currently some 20 such programs can be found on the Internet (Figure 4), differing considerably in their size, easy of use, development status and licensing. An incomplete list includes ACD/SDA [8], ChemWriter [9], Edit2D [10], ensochemEditor [11], ICedit [12], JavaGrins [13], JME [14], JUME [15], KegDraw [16], Marvin Sketch [17], Osiris [18] and Symyx JDraw [19]. Additionally, several Java editors are available also in open source, including JChemPaint [20], JMolDraw [21], MCDL [22] and SketchEl [23], encouraging collaborative software development by the global cheminformatics community.

In the following section the JME Molecule Editor will be described in more details, as a representative example of this type of web-based structure input tools.

## JME Molecule Editor

**Figure 5:**
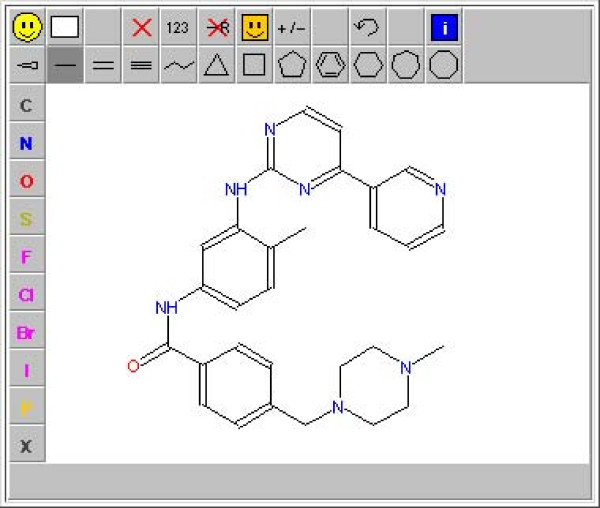
**JME Molecule Editor**.

The JME Molecule Editor [14] is a Java applet which allows one to draw, edit, and display molecules and reactions directly within web page (Figure 5). The editor was originally written at Comenius University in Bratislava in QuickBASIC by author of this review and later translated into Java to be used as a structure input tool for an in-house web-based cheminformatics system at Ciba-Geigy and later at Novartis [5,24]. Due to many requests, the JME editor has been released to the public and is currently probably the most popular molecular entry system on the web.

**Figure 6:**
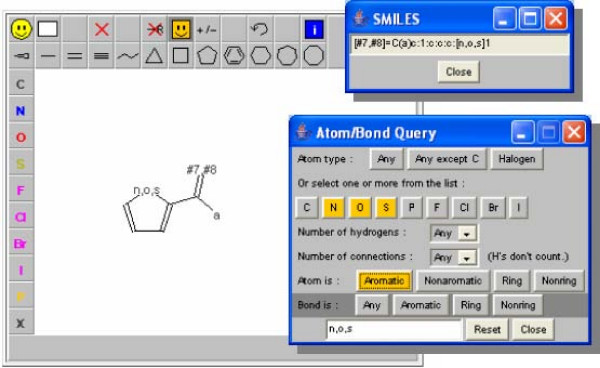
**Creation of substructure queries with the JME**.

**Figure 7:**
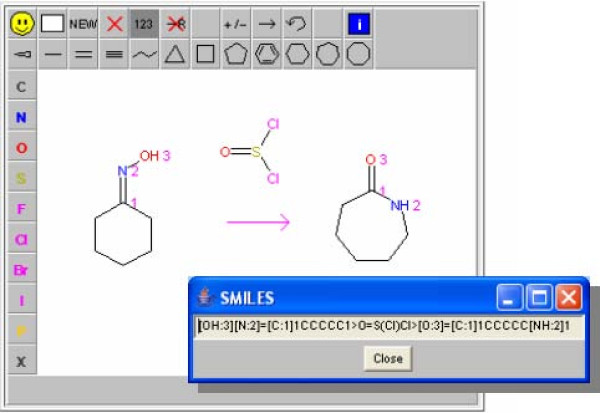
**Input of reactions with the JME**.

The JME editor is able to generate SMILES, MDL Molfile or its own compact format (one line textual representation of a molecule or reaction including also atomic 2D coordinates) of the input molecules. The SMILES code generated by JME is canonical, i.e. independent on the way how the molecule was drawn. The applet can also serve as a query input tool for searching molecular databases by supporting creation of complex substructure queries (Figure 6), which are automatically translated into SMARTS [25]. With help of simple HTML form elements, the creation of 3D structure queries is also possible, as used for example in 3D pharmacophore searches in the NCI database system [26]. Input of reactions is also supported (Figure 7), including generation of reaction SMILES and SMIRKS [27].

The JME applet can communicate with other elements on the HTML page via its public functions. These functions allow one to retrieve created molecules, change JME appearance or programmatically display new molecules. JME can be, for example, easily connected with a graph on a web page and when mouse touches a point on the graph, display the corresponding molecule (as shown for example here [28]). JavaScript functions, called automatically when a web page containing JME is loaded or unloaded, allow the archiving of the current molecule into a cookie (a small piece of persistent information associated with this particular web page stored on the client computer) and the display of the saved molecule when the web page is loaded again. Detailed description of JME public functions is available on-line in the JME documentation [14].

The JME applet is written in Java 1.0, the initial version of Java programming language. Java 1.0 does not support any sophisticated graphics or user interface elements, but on the other side, it is available in all types and versions of web browsers, therefore JME can run practically everywhere, without the need to rely on additional Java plugins (which are not always available). Another advantage of the simple architecture is the JME size (below 40 kB), which assures very fast loading in web pages. Thank to the Java platform independence, the JME runs on Windows PCs, Mac/OS machines and practically all UNIX clones, including, of course, LINUX.

The number of Internet sites which use the JME applet as a molecule input tool is too large to be listed here. These sites include molecular databases, property prediction services, various cheminformatics tools (such as generation of 3D structures, prediction of metabolic sites, combinatorial library design, or QSAR services just to name some) or interactive sites focused on chemistry education. Several such representative applications, which may be of general interest, have been collected here [29]. For an additional review of free web tools focused on applications in medicinal chemistry and drug discovery, see review [30].

For non-commercial purposes, the JME Editor may be obtained directly from the author of this review.

## Server Side Editors

As shown in the previous sections, Java applets offer a very useful way to add additional functionality to web pages. But the applets have also disadvantages. The major one is the dependency on the Java runtime engine. This may be a problem particularly in the industry environment, where sometimes Java or communication between applets and JavaScript in web browsers, is blocked for security reasons. Additionally, the many possible combinations of browsers, operating systems and various Java versions make development of more complex applets, particularly those relying on the latest Java features, rather challenging and far from vision of the Java slogan, "write once run anywhere". This suboptimal situation, a relic from so called "browser wars" (hard competition for dominance in the browser market when incompatibility with standards or other browsers was sometimes seen as a way to gain market control), is the reason why one can see the return of server-side molecule editors with light JavaScript clients and the actual molecule processing done on the server. Unlike the early editors based on clickable maps and cgi scripts, the current server side editors use modern technologies like image streaming or Ajax for communication with the server, making this process much faster. The advantage of server-side editors is that they are fully platform independent, relying only on a JavaScript engine, which is available in practically all browsers.

**Figure 8:**
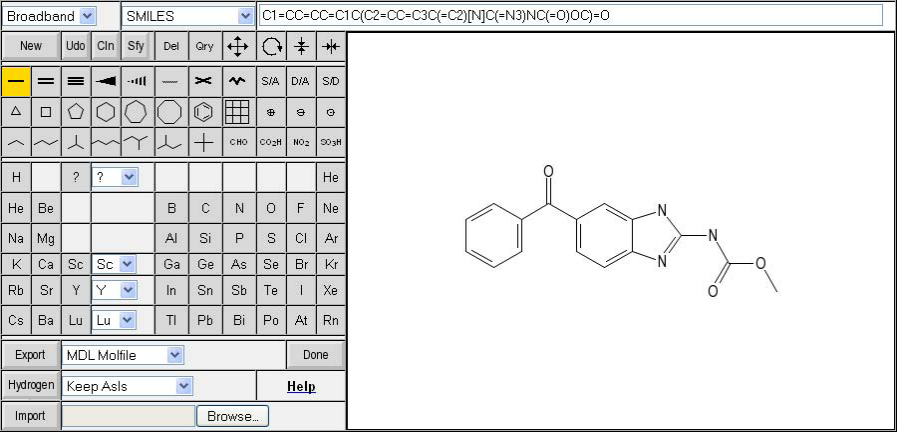
**The PubChem Chemical Structure Sketcher**.

The first server side editor of this new generation was the PubChem Chemical Structure Sketcher [31] (Figure 8), developed as a structure input tool for querying the popular PubChem database [32]. The editor consists of couple of HTML pages with embedded JavaScript functions, and FastCGI server script powered by the Cactvs toolkit. In response to mouse or key actions, the server generates and sends back image streams several times per seconds to update the editor drawing area. To insure the necesary robustness needed when supporting the highly used PubChem database the sketcher uses two independent multi-processor server hosts and redundant database servers for storing its state.

**Figure 9:**
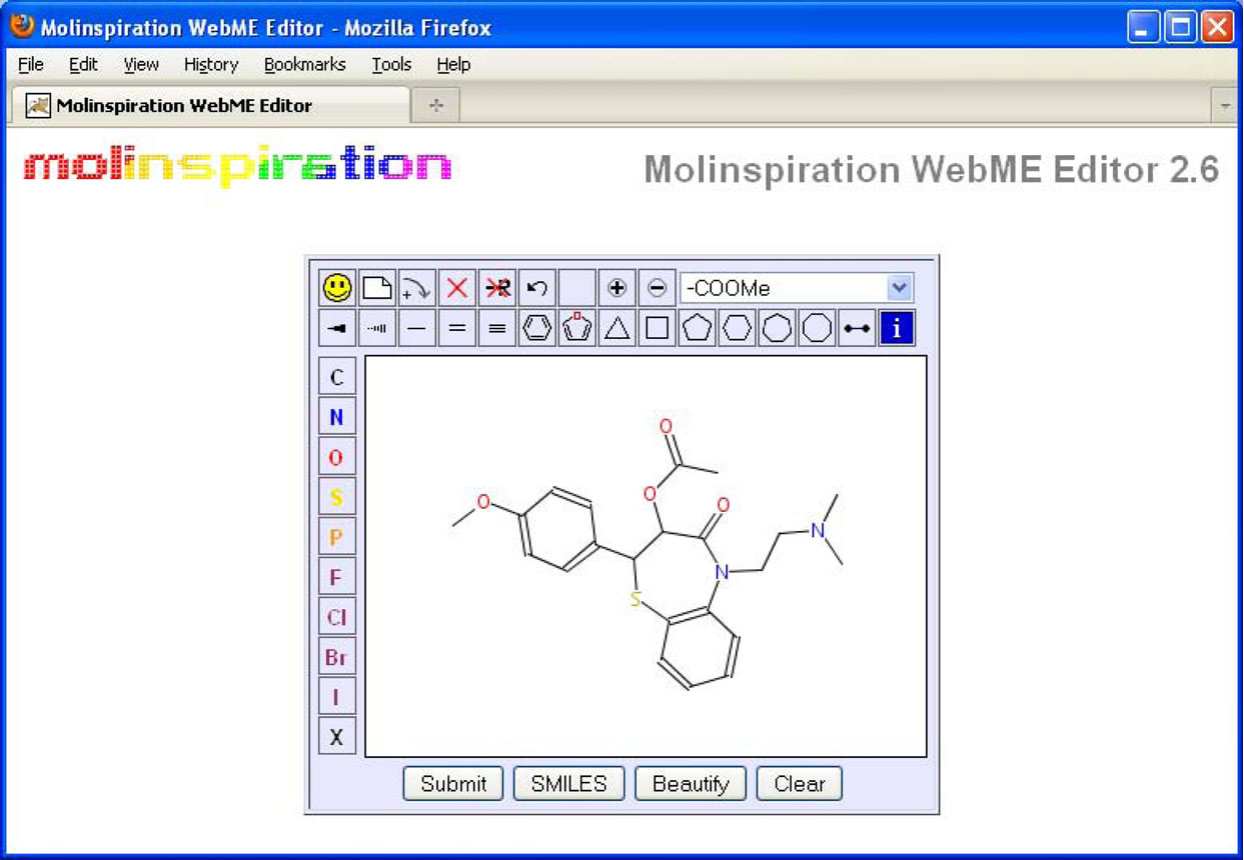
**WebME Ajax Editor from Molinspiration**.

Another server side editor is WebME [33] from Molinspiration (Figure 9). WebME has a lightweight GUI based on the JQuery JavaScript toolkit with mouse movement detection. Advantage of this approach is that the GUI offers a visual clue when an atom or bond is touched, so the user experience is similar to those when using an interactive applet. WebME client communicates with its server using the Ajax (Asynchronous JavaScript and XML) technology. The server side molecular processing engine is powered by a servlet, with a resulting fast response time.

## Future of the Molecule Editing on the Web

The new paradigm of web computing, namely the web browser taking over the function of operating system itself, accompanied by fast progress in performance of JavaScript engines, encourages development of scientific applications running entirely within web browsers. This is facilitated also by introduction of a new canvas element in the HTML5 standard, that allows dynamic drawing on web pages using only JavaScript functions. Also the introduction of Google Web Toolkit (GWT), a development environment that allows easy creation of interactive web application in Java and then translates the Java code into the optimized JavaScript, makes development of rich JavaScript applications much easier. As a consequence of this technology progress, several molecule editors relying purely on JavaScript have become available, for example jsMolEditor [34] or WebCME [35]. These editors are presently still in development stage, but clearly show the potential of JavaScript programming connected with browser-based graphics. Several other projects using JavaScript to develop web-based structure input tools are ongoing, including also an effort of the author of this review to translate the JME Java applet to JavaScript [36]. Another interesting cheminformatics JavaScript application is a collection of components called ChemDoodle [37]; this includes 2D and 3D molecule viewers, and also a simple molecule editor called Doodler.

Despite all advantages of JavaScript, however, one needs to be aware also of disadvantages of this technology. The most important is lack of support of the canvas element in older browsers, particularly in the Internet Explorer (although Internet Explorer 9 should have canvas support). Another limitation of client-side JavaScript programs is requirement for fast loading, which sets restrictions to their size and therefore also to the available functionality (for comparison the server code of the PubChem Sketcher, that supports many complex features, supports dozens of file formats and is capable to re-layout complex drawings, has size of about 45 Mb).

In summary, JavaScript based molecule editors, and generally all cheminformatics applications based on this technology have great potential, but it will still take some time until the canvas graphics is globally supported and resource intensive JavaScript applications will be able to run smoothly in all browsers.

Another technology for adding interactivity to web pages is Adobe Flash. Although currently used mostly for creating advertisements and video-streamimg applications, integrated scripting language called ActionScript also allows interactive drawing within web pages. To the authors knowledge, no molecule editor has been written in Flash, yet, but available chemical applications like chemical structure viewer [38], or crystal viewer [39], show that that Flash can be used to create nice interactive chemical applications. One can therefore expect that sooner or later, a molecule editor written in Flash will appear.

## Conclusions

This short overview of history and current status of web-based structure input tools illustrates clearly that the web technology is evolving extremely quickly. New technologies, new tools and services are appearing almost daily. Web-based cheminformatics applications are following this trend. It is also gratifying to see the advent of open source movement in cheminformatics on the Internet, as advocated for example by the Blue Obelisk Group [40] and witnessed by collaborative projects like Chemistry Development Kit CDK [41], Jmol [42], Bioclipse [43] and several others. In this respect, cheminformatics seems to be closing the gap on the traditionally more open source world of bioinformatics. The future of cheminformatics, and particularly of cheminformatics applications on the web, is indeed exciting.

## Competing interests

The author declares that they have no competing interests.
